# Supplementary material for: Neuron Compatibility and Antioxidant Activity of Barium Titanate and Lithium Niobate Nanoparticles
Source: Int J Mol Sci. 2022 Feb 3;23(3):1761. doi: 10.3390/ijms23031761 (PMC8836423; doi:10.3390/ijms23031761)
Supplement: Supplementary file 1 [file ijms-23-01761-s001.zip › ijms-1530389-supplementary.pdf]

International Journal of *Molecular Sciences*

MDPI

Supplementary material

Neuron compatibility and antioxidant activity of barium titanate and lithium niobate nanoparticles

Mariarita Candito[1,2], Edi Simoni[1,2], Erica Gentilin[1,2], Alessandro Martini[1,2,3], Gino Marioni[1,4,*], Serena Danti[2,5,6], Laura Astolfi[1,2,4*]

[1] Bioacoustics Research Laboratory, Department of Neurosciences, University of Padova, via G. Orus, 2b, 35129 Padova, Italy. mariarita.candito@phd.unipd.it, edi.simoni@unipd.it, erica.gentilin@unipd.it, alessandromartini@unipd.it, gino.marioni@unipd.it, laura.astolfi@unipd.it

[2] National Interuniversity Consortium of Materials Science and Technology (INSTM), via G. Giusti 9, 50121 Firenze, Italy serena.danti@unipi.it

[3] I–APPROVE, International Auditory Processing Project in Venice, Department of Neurosciences, University of Padova, Santi Giovanni e Paolo Hospital, ULSS3 Serenissima, Venezia, Italy

[4] Otolaryngology Unit, Department of Neuroscience DNS, University Hospital of Padova, via Giustiniani, 2, 35129 Padova, Italy

[5] Department of Civil and Industrial Engineering, University of Pisa, Largo Lucio Lazzarino, 56126 Pisa, Italy

[6] Department of Civil and Environmental Engineering, Massachusetts Institute of Technology (MIT), Massachusetts Ave. 77, Cambridge, Massachusetts 02139, USA

* Correspondence: gino.marioni@unipd.it; laura.astolfi@unipd.it; Tel.: +39 049 8212029 (G.M.)

Citation: Candito, M.; Simoni, E.; Gentilin, E.; Martini, A.; Marioni, G.; Danti, S.; Astolfi, L. Neuron Compatibility and Antioxidant Activity of Barium Titanate and Lithium Niobate Nanoparticles. *Int. J. Mol. Sci.* **2022**, *23*, 1761. https://doi.org/10.3390/ijms23031761

Academic Editor: Rolf Heumann

Received: 13 December 2021
Accepted: 1 February 2022
Published: 3 February 2022

Publisher's Note: MDPI stays neutral with regard to jurisdictional claims in published maps and institutional affiliations.

Copyright: © 2022 by the authors. Licensee MDPI, Basel, Switzerland. This article is an open access article distributed under the terms and conditions of the Creative Commons Attribution (CC BY) license (http://creativecommons.org/licenses/by/4.0/).

The morphology of the plain $BaTiO_3$ and $LiNbO_3$ nanoparticles was evaluated by scanning electron microscopy (SEM) using a FEI FEG-Quanta 450 instrument (Field Electron and Ion Company, Hillsboro, Oregon, USA). The samples were sputter-coated with Gold (Gold Edwards SP150B, England) before analysis. SEM micrographs were acquired at different magnifications to visualize the details of interest. The highest magnifications are reported in Figure S1, in which submicrometric size is confirmed.

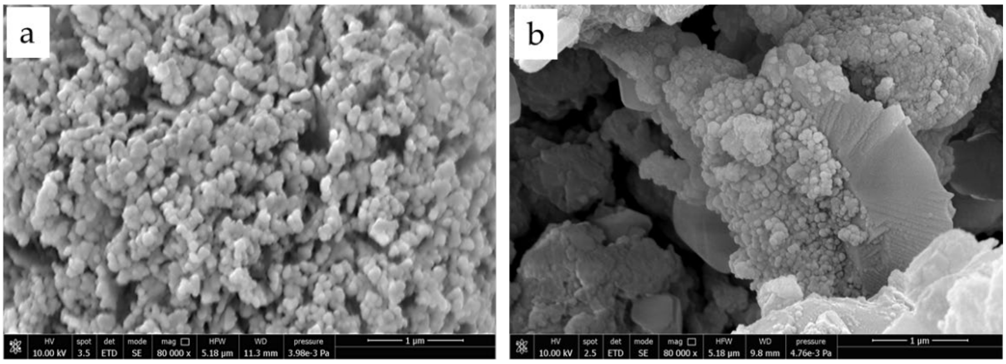

Figure S1. Results of SEM analysis of pristine (a) barium titanate ($BaTiO_3$) and (b) lithium niobate ($LiNbO_3$) at 80,000× magnification. (b):*"Reprinted with permission n. 5236510541378 from: Danti, S., Azimi, B., Candito, M., Fusco, A., Sorayani Bafqi, M.S., Ricci, C., Milazzo, M., Cristallini, C., Latifi, M., Donnarumma, G., Bruschini, L., Lazzeri, A., Astolfi, L., Berrettini, S., 2020. Lithium niobate nanoparticles as biofunctional interface material for inner ear devices. Biointerphases. 15, 031004. Copyright 2020, American Vacuum Society."*
